# Baseline Corrected QT Interval Dispersion Is Useful to Predict Effectiveness of Metoprolol on Pediatric Postural Tachycardia Syndrome

**DOI:** 10.3389/fcvm.2021.808512

**Published:** 2022-01-20

**Authors:** Yuanyuan Wang, Yan Sun, Qingyou Zhang, Chunyu Zhang, Ping Liu, Yuli Wang, Chaoshu Tang, Hongfang Jin, Junbao Du

**Affiliations:** ^1^Department of Pediatrics, Peking University First Hospital, Beijing, China; ^2^Key Lab of Molecular Cardiovascular Sciences, Ministry of Education, Beijing, China; ^3^Department of Physiology and Pathophysiology, Health Science Centre, Peking University, Beijing, China

**Keywords:** corrected QT interval dispersion, postural tachycardia syndrome, metoprolol, orthostatic intolerance, children

## Abstract

**Objectives:**

The study was designed to explore the role of baseline-corrected QT interval dispersion (QTcd) in predicting the effectiveness of metoprolol on pediatric postural tachycardia syndrome (POTS).

**Methods:**

There were two groups in the study, the discovery group and the validation group. The children with POTS in the discovery group were treated with oral metoprolol, with the completed necessary medical records, head-up tilt test (HUTT), blood chemistry, and 12-lead ECG before treatment at the pediatrics of Peking University First Hospital, China. According to whether the symptom score (SS) was reduced by more than 2 points after administration with oral metoprolol as compared with that before treatment, the children with POTS were separated into responders and non-responders. The demographic characteristics, hemodynamic indicators, and the QTcd of the two groups were compared, and the estimate of the baseline QTcd in predicting the treatment response to metoprolol was tested through a receiver operating characteristic (ROC) analysis. Other 24 children suffering from POTS who were, administrated with metoprolol at the pediatrics of Peking University First Hospital were included in the validation group. The sensitivity, specificity, and accuracy of the baseline QTcd in the prediction of the effectiveness of metoprolol on POTS were validated in children.

**Results:**

The pre-treatment baseline QTcd in responders treated with metoprolol was longer than that of the non-responders in the discovery group [(66.3 ± 20.3) ms vs. (45.7 ± 19.9) ms, *p* = 0.001]. The baseline QTcd was negatively correlated with SS after metoprolol treatment (*r* = −0.406, *p* = 0.003). The cut-off value of baseline QTcd for the prediction of the effectiveness of metoprolol on pediatric POTS was 47.9 ms, yielding a sensitivity of 78.9% and a specificity of 83.3%, respectively. The validation group showed that the sensitivity, specificity, and accuracy of the baseline QTcd ≥ 47.9 ms before treatment for estimating the effectiveness of metoprolol on POTS in children were 73.7, 80.0, and 75.0%, respectively.

**Conclusion:**

Baseline QTcd is effective for predicting the effectiveness of metoprolol on pediatric POTS.

## Introduction

Postural tachycardia syndrome (POTS), as one of the clinical subtypes of orthostatic intolerance (OI), mainly manifests an increase in heart rate (HR) in the standing position and OI symptoms. It greatly influences the quality of life of children both physically and mentally (1, 2). Its main pathogenesis includes high catecholamine levels in blood circulation, overexcitement of sympathetic nerve function, vasodilation, or low central blood volume (3–6). Beta-adrenoceptor blocker metoprolol was currently one of the main drugs for the treatment of children with POTS. Metoprolol exerts its therapeutic effect on POTS in children by inhibiting excessive sympathetic nerve function, decreasing HR, and the mechanical activation of ventricular wall baroreceptors, and inhibiting the effect of excessive plasm catecholamine content (7–10). However, the effective rate of metoprolol on POTS in children ranged from 57.1 to 57.89% (11). The main reason was that beta-adrenoceptor blockers could ameliorate the symptoms only for POTS children whose pathogenesis consisted of high catecholamine levels (11). It could be inferred that if POTS children whose pathogenesis involves high circulating catecholamine levels could be predicted before treatment, they could be given a targeted treatment of beta-adrenoceptor blockers, which would significantly improve the efficacy of POTS in children. Therefore, it is of great important significance to find out the useful, easy-to-perform, and inexpensive indicators or markers to predict the effectiveness of beta-adrenoceptor blocker in children suffering from POTS before treatment.

Previously, the investigators examined the baseline values of plasma norepinephrine (12), copeptin (13), C-type natriuretic peptide (CNP) levels (14), and 24-h heart rate variability (HRV) in predicting the efficacy of metoprolol on pediatric POTS (15), and then guided the clinical use of metoprolol in the treatment with the useful markers detected. However, the plasma norepinephrine is extremely variable, which limits the accuracy of the prediction. The detection of plasma copeptin requires enzyme-linked immunosorbent assay, the operation process of which is complicated, limiting the popularization of the technique for general practitioners. In addition, the determination of plasma norepinephrine, copeptin, and CNP levels is invasive because the blood samples are obtained by venipuncture. The collection and recording of 24-h HRV are time-consuming and non-economic. As such, all the above-mentioned predictive techniques have obvious limitations in clinical application. Therefore, looking for stable, non-invasive, and easy-to-perform indicators before treatment to predict the efficacy of metoprolol on POTS in children is a critical issue in this field.

The previous reports showed that the corrected QT interval dispersion (QTcd) obtained by 12-lead ECG could quantitatively assess the activity of autonomic nerve function, which was non-invasive, easier to operate, and inexpensive (15, 16). Accordingly, the present study was designed to reveal if the baseline QTcd was useful in predicting the efficacy of metoprolol on POTS in children with POTS.

## Materials and Methods

### Population

In this retrospective cohort study, there were 53 children suffering from POTS who were hospitalized in the Department of Pediatrics, Peking University First Hospital, Beijing, China from March 8, 2012 to August 3, 2018, with complete data of 12-lead ECG prior to the treatment and treated with metoprolol. Furthermore, 3 months after the first metoprolol therapy, telephonic, outpatient, or inpatient follow-up was conducted. The information of 3 children with POTS was missing during the follow-up or had incomplete follow-up data. The lost follow-up rate was 5.7%. Therefore, this study finally included 50 children with POTS treated with oral metoprolol in the discovery group [28 boys and 22 girls, mean age of 12.5 ± 2.1 years].

**Figure 1:**
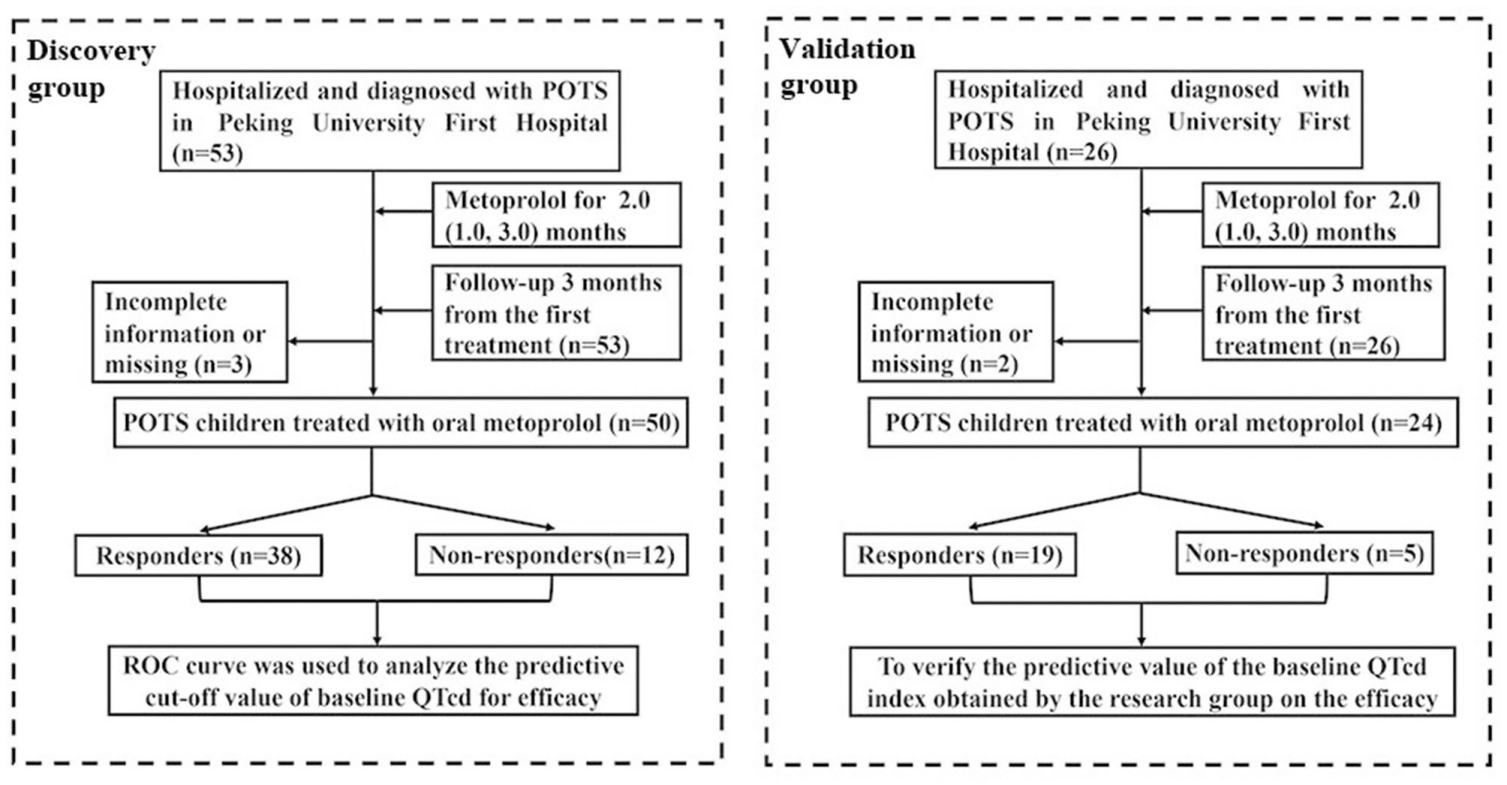
Flowchart of inclusion of participants in this study. QTcd, corrected QT interval dispersion; POTS, postural tachycardia syndrome.

In addition, 26 children with POTS, who had completed 12-lead ECG data before treatment and treated with metoprolol in the Department of Pediatrics, Peking University First Hospital from August 31, 2018 to December 10, 2020, were recruited in the validation group. After 3 months of the treatment with metoprolol, the outpatient or inpatient follow-up was conducted, and 2 cases of children were lost to follow-up or with incomplete data in follow-up. The loss rate of follow-up was about 7.7%. Therefore, 24 children with POTS were finally included in the treatment with oral metoprolol in the validation group [9 boys and 15 girls, at a mean age of 11.9 ± 2.1 years]. Figure 1 shows the flowchart for the subject inclusion in this investigation.

This research was granted by the ethics committee of the Peking University First Hospital [2018(202)]. The guardians or parents of the children were notified of the study aim and were requested to sign an informed consent form.

### Inclusion Criteria of Children

The inclusion criteria are: children with POTS who were hospitalized in Peking University First Hospital; the clinical manifestations after admission, complete 12-lead ECG examination data, and necessary laboratory investigations before metoprolol treatment were all recorded; the age range was 6–18 years old; and the children with POTS were treated with oral metoprolol.

### Diagnostic Criteria for POTS in Children

Children with POTS were often accompanied by quick changes in body position (from lying to standing), persistent standing, muggy environment, and other predisposing factors. They had OI symptoms, such as dizziness, headache, palpitations, and syncope. During the standing test or basic head-up tilt test (BHUTT), within 10 min from supine to the standing, the maximum heart rate (HR) when standing increases by 40 bpm compared with that of the supine position or the maximum of HR (HR max) within 10 min of upright position reaches the standard (≥ 130 bpm for 6–12 years old, or ≥ 125 bpm for 13–18 years old), and the blood pressure (BP) drops <20/10 mmHg (17, 18).

### Exclusion Criteria for POTS in Children

Patients suffering from cardiovascular disorders, such as arrhythmia, myocarditis, cardiomyopathy, pulmonary hypertension, and pseudo-syncopal diseases, such as metabolic diseases, psychogenic pseudoscope, or epilepsy, were excluded. In addition, children with incomplete data of 12-lead ECG before treatment and children who were lost to follow-up after metoprolol treatment were excluded.

### Standing Test

The test requires a suitable temperature, dim light, and quiet environment. First, the patients were required to rest for 10 min in the supine position, and then to stand actively for 10 min. During the examination, ECG, HR, and BP were consecutively recorded with a multi-channel physiological monitor (Dash 2000, General Electric Company, NY, USA). During the standing process, if the child had syncope or pre-syncope, the test was terminated and then immediately the child was assisted to a supine position (19).

### Basic Head-Up Tilt Test

All patients were asked to terminate the medicine that affects autonomic nerve function for longer than 5 half-life time prior to the test, and needed to fast for over 4 h. Under a noiseless, suitable temperature, and low-light condition, the child was required to lie supine on a tilted bed (HUT-821, Juchi Company, Beijing, China) for 10–30 min. And HR and ECG were constantly recorded using a multi-lead ECG monitor (General Electric, NY, USA). Continuous BP was recorded by a non-invasive arterial blood pressure monitoring system (Finapres Medical System, Finometer PRO, FMS company, the Netherlands). When HR, BP, and ECG were all stable, the inclined bed started to raise with an angle of 60 degrees, and HR, BP, and ECG were recorded till the whole process of the BHUTT was completed (20).

### Twelve-Lead ECG Examination and QTcd Measurement

Before treatment, all children received a 12-lead ECG test with an electrocardiograph (FX-7402, Fukuda, Japan). The children were required to lie and have a rest for about 10 min in the supine position, keep breathing steady, and take a regular 12-lead continuous tracing. The paper speed of ECG was set to 25 mm s^−1^, and the amplitude to 10 mm (mV)^−1^. After that, the stable and clear baseline ECG graph was recorded. And QT interval was measured manually by a full-time researcher.

The QT interval stands for the interval from the starting point of QRS wave to the end of T wave. The average of 3 consecutive QT intervals in each lead was obtained, and the maximum QT interval (QTmax) and the minimum QT interval (QTmin) were measured in the 12-lead ECG. In addition, the maximum RR interval (RRmax) and the minimum RR interval (RRmin) were calculated. Then, the QT dispersion (QTd) was calculated as QTd = QTmax—QTmin. Additionally, the corrected QTmax (QTcmax) and corrected QTmin were calculated using the Bazetts formula. The specific calculation was performed as QTcmax = QTmax ÷ RRmax^1/2^ and QTcmin = QTmin ÷ RRmin^1/2^. The calculation formula of corrected QTd (QTcd) was QTcd = QTcmax—QTcmin.

### Symptom Score in Children With POTS

Symptom score (SS) is based on the frequency of the OI symptoms, which are as follows: dizziness, syncope, headache, chest tightness, palpitation, sweating, nausea, hand tremor, blurred vision, and inattention. The scoring criteria of any symptom frequency were as follows (21–25): 0 score stands for no symptom; 1 score, symptoms once a month; 2 scores, symptoms 2–4 times per month; 3 scores, symptoms 2–7 times per week; 4 scores, symptoms > once a day. The score of each symptom was recorded, and the total SS was the sum of the scores of each symptom for a child.

### Treatment and Follow-Up

After all children in the study were first diagnosed with POTS, they were treated with metoprolol for 2 (1, 3) months. The initial dosage of metoprolol was used as 0.5 mg·kg^−1^·day^−1^, two times a day, and it was increased gradually, according to the tolerable dose (the maximum dose is 2 mg·kg^−1^·day^−1^).

All subjects suffering from POTS were followed-up for a period of 3 months after the treatment with metoprolol and it was recorded by the trained personnel. In addition, questionnaire surveys were conducted through outpatient, in-patient, and telephonic follow-up. The drug compliance, frequency of OI symptoms, and drug adverse effects should be carefully monitored during the follow-up.

### Grouping Based on Therapeutic Effect of Metoprolol at Follow-Up

When POTS was first diagnosed in children, SS was calculated for the patients as the pre-treatment SS. Post-treatment SS was calculated after the follow-up.

If the post-treatment SS of the child was decreased by ≥2 scores as compared with the pre-treatment SS, the child was defined as a “responder.” On the contrary, when post-treatment SS was decreased by <2 scores, the child was defined as a “non-responder” (22–24).

The POTS children in the validation group were divided into “predicted responders” and “predicted non-responders,” according to the baseline QTcd prediction cut-off value obtained by the discovery group. According to the actual follow-up results, the children in the validation group were divided into “actual responders” and “actual non-responders.” Thereby, the sensitivity, specificity, and accuracy of baseline pre-treatment QTcd in estimating the effectiveness of metoprolol on POTS in children were validated.

### Statistical Analysis

The statistical data analysis software used in this study is SPSS 23.0 (IBM, Armonk, NY, USA). The measurement data are represented by mean ± SD, and the count data are represented by the number of cases (*n*). The Shapiro–Wilk test was used by the normality test of measurement data. When the data in the two groups were in a normal distribution, the independent *t*-test was utilized for the comparison between the groups, otherwise, the Mann–Whitney *U*-test was used. The χ^2^-test was applied for comparing count data between the groups. Pearson analysis was applied to test the correlation between baseline QTd or QTcd and post-treatment SS. A receiver operating characteristic (ROC) curve was applied to estimate the cut-off values of baseline QTd or QTcd in predicting the effectiveness of metoprolol on pediatric POTS in the discovery group. When the maximum value of the Youden index was taken, its predictive sensitivity and specificity reached the best. Therefore, according to the outcome of the 3-month-follow-up after metoprolol treatment, the QTcd cut-off value from the discovery group was calculated. The sensitivity, specificity, and accuracy of baseline QTcd to predict the therapeutic efficacy were verified in the validation group. The standard for statistically significant differences was *p* < 0.05.

## Results

### Analysis of Demographic Characteristics, Baseline Hemodynamic Indexes, and Pre-treatment SS Between Responders and Non-responders in the Discovery Group

There were no marked differences in gender, age, weight, height, body mass index (BMI), HR, systolic blood pressure (SBP), and diastolic blood pressure (DBP) in the supine position, HR max, ΔHR, and pre-treatment SS between 38 responders [20 boys (52.6%), mean age (12.4 ± 2.3) years] and 12 non-responders [8 boys (66.7%), the mean age (12.8 ± 1.5) years] in discovery group (all *p* > 0.05; Table 1).

**Table 1:** Comparison of demographic and hemodynamic parameters between responders and non-responders in the discovery group.

**Groups**	**Cases [*n* (%)]**	**Sex (M/F)**	**Age (yrs)**	**Height (cm)**	**Weight (kg)**	**BMI (kg/m^**2**^)**	**Supine HR (bpm)**	**Supine SBP (mmHg)**	**Supine DBP (mmHg)**	**ΔHR bpm**	**HR max (bpm)**	**Pre-Treatment SS (points)**
Responders	38 (76.0)	20/18	12.4 ± 2.3	159.3 ± 15.5	53.4 ± 20.5^a^	20.3 ± 4.9^a^	72 ± 14	110 ± 17^a^	70 ± 16	47 ± 7	123 ± 10	7 ± 4^a^
Non-responders	12 (24.0)	8/4	12.8 ± 1.5	162.3 ± 7.5	55.9 ± 17.9	20.9 ± 5.2	76 ± 15	110 ± 12	65 ± 11^a^	47 ± 8^a^	123 ± 12	9 ± 5
t/Z/χ^2^	–	0.271	0.591	0.927	−0.466	−0.454	0.797	−0.057	−0.841	−0.421	−0.170	−1.759
*P*-Value	–	0.603	0.557	0.359	0.641	0.650	0.429	0.955	0.400	0.674	0.866	0.079

*POTS, postural tachycardia syndrome; M/F, male/female; BMI, body mass index; kg/m^2^, kilogram per square meter; HR, heart rate; bpm, beats per minutes; SBP, systolic blood pressure; DBP, diastolic blood pressure; ΔHR, the difference between the maximum heart rate within 10 min of standing and the supine position; HR max, maximum heart rate within 10 min of standing; SS, symptom score*.

*Data are mean ± SD*.

a *Non-normal distribution*.

### Analysis of Baseline ECG Indexes Between the Responders and the Non-responders in the Discovery Group

**Figure 2:**
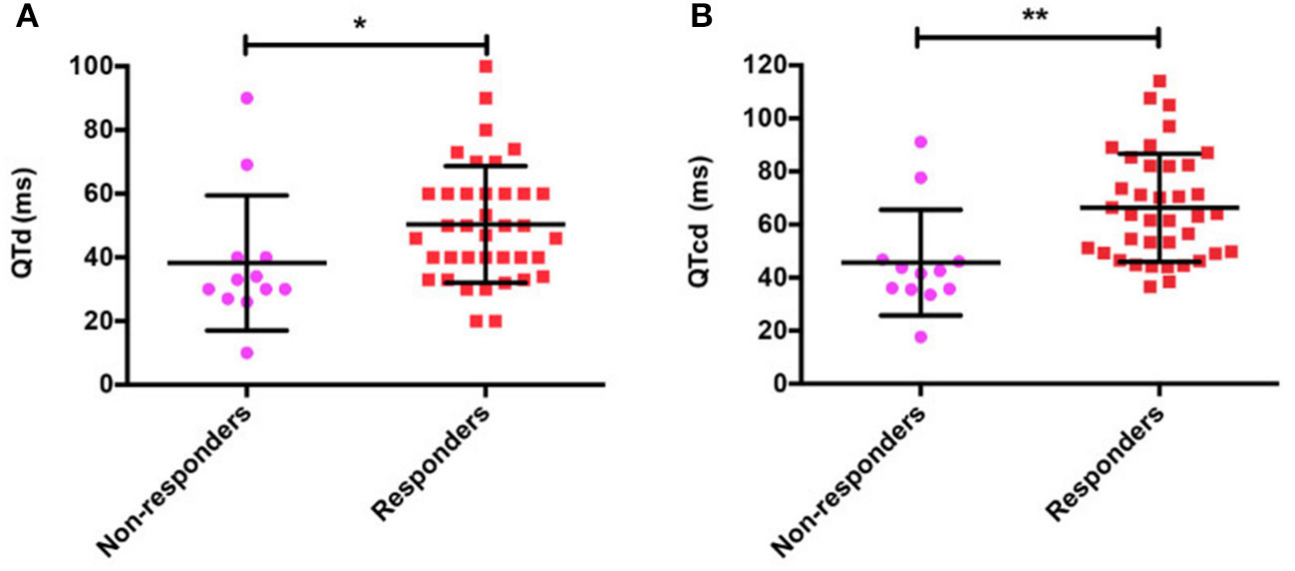
QT interval dispersion (QTd) **(A)** and corrected QT interval dispersion (QTcd) **(B)** in responders and non-responders with POTS treated with metoprolol. POTS, postural tachycardia syndrome; QTd, QT interval dispersion; QTcd, corrected QT interval dispersion; ms, millisecond, *p < 0.05; **p < 0.01.

The comparison of baseline ECG indexes revealed that there were no obvious differences in pre-treatment baseline QTmax, RRmax, RRmin, QTcmax, and QTcmin between responders and non-responders (all *p* > 0.05; Table 2). While the pre-treatment QTmin in the responders was shorter than the non-responders [(330.5 ± 26.8) ms vs. (357.5 ± 26.9) ms, *t* = 3.034, *p* = 0.004]. Furthermore, the baseline QTd (Figure 2A) and QTcd (Figure 2B) in the responders were markedly longer than the non-responders [(50.4 ± 18.3) ms vs. (38.3 ± 21.2) ms, *Z* = −2.455, *p* = 0.014; (66.3 ± 20.3) ms vs. (45.7 ± 19.9) ms, *Z* = −3.339, *p* = 0.001].

**Table 2:** Comparison of baseline ECG parameters between responders and non-responders in the discovery group.

**Groups**	**QTmax (ms)**	**QTmin (ms)**	**RRmax (ms)**	**RRmin (ms)**	**QTcmax (ms)**	**QTcmin (ms)**
Responders	380.4 ± 26.7	330.5 ± 26.8	771.6 ± 127.0	812.6 ± 130.1	435.1 ± 22.0	368.8 ± 28.1
Non-responders	395.8 ± 23.7	357.5 ± 26.9	841.1 ± 122.2	859.7 ± 128.0	433.7 ± 24.0	388.0 ± 33.4
*t*	1.789	3.034	1.734	1.096	−0.199	1.966
*P*-Value	0.080	0.004	0.089	0.279	0.843	0.055

*POTS, postural tachycardia syndrome; ms, millisecond; QTmax, the maximum value of QT interval in 12 leads of ECG; QTmin, the minimum value of QT interval in 12 leads of ECG; RRmax, the maximum value of RR interval in 12 leads of ECG; RRmin, the minimum value of RR interval in 12 leads of ECG. Data are mean ± SD*.

### Correlation Analysis Between Baseline QTd and QTcd and Post-treatment SS in the Discovery Group

**Figure 3:**
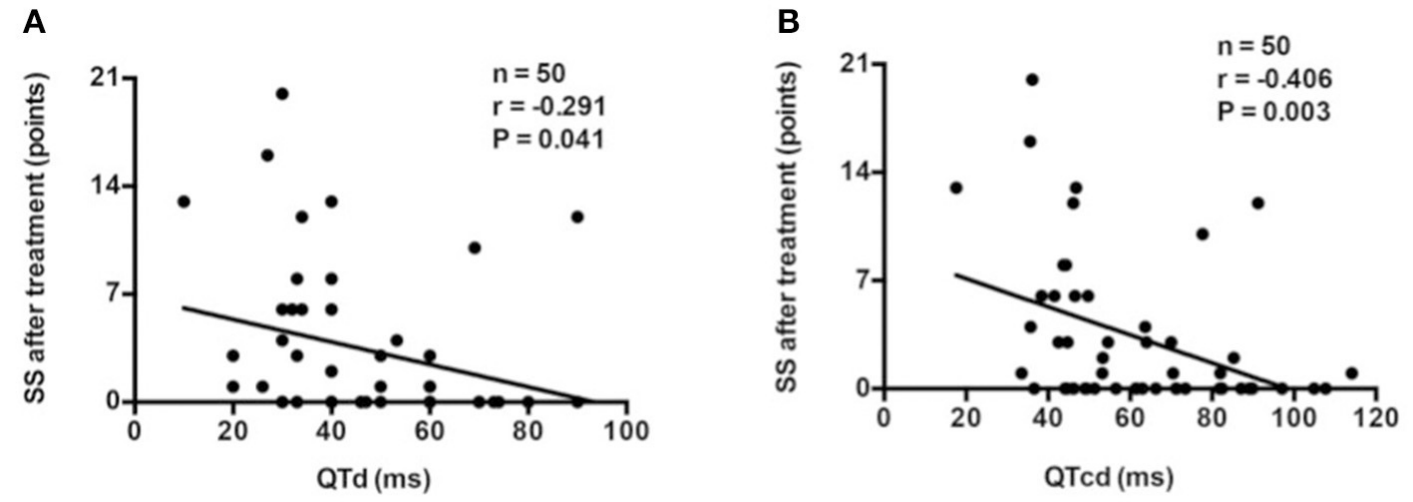
Correlation analysis between baseline QTd **(A)** or QTcd **(B)** and SS after treatment in the discovery group. QTd, QT interval dispersion; QTcd, corrected QT interval dispersion; SS, symptom score.

Pre-treatment QTd (*r* = −0.291, *p* = 0.041; Figure 3A) or QTcd (*r* = −0.406, *p* = 0.003; Figure 3B) was negatively correlated with post-treatment SS.

### ROC Analysis of Pre-treatment Baseline QTd and QTcd in the Prediction of Efficacy of Metoprolol

**Figure 4:**
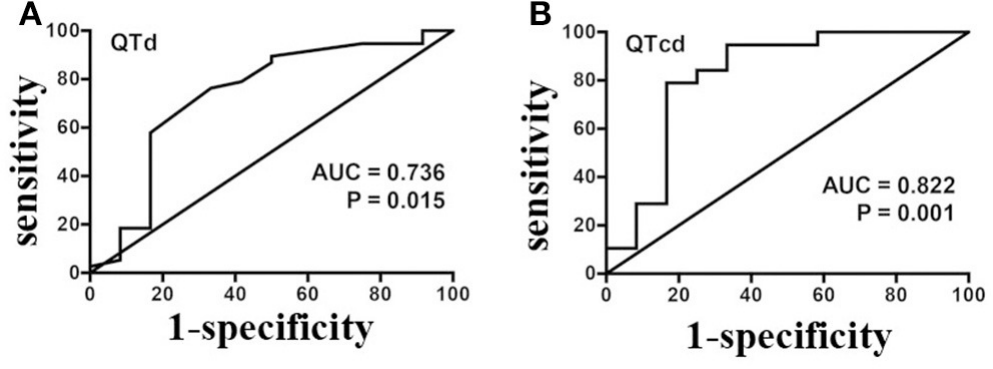
A ROC analysis of baseline QTd **(A)** and QTcd **(B)** in evaluating the effectiveness of metoprolol in POTS of children in the discovery set. ROC, receiver operating characteristic curve; POTS, postural tachycardia syndrome; QTd, QT interval dispersion; QTcd, corrected QT interval dispersion; AUC, area under the curve.

Since the baseline QTd and QTcd significantly differed between the responders and non-responders, the value of predicting the efficacy of metoprolol on POTS children was further analyzed with an ROC curve. The results demonstrated that the area under the curve (AUC) of baseline QTd (Figure 4A) and QTcd (Figure 4B) in predicting the efficacy of metoprolol on POTS in children was 0.736 [95% CI 0.552–0.920] and 0.822 (95% CI 0.653–0.992), respectively. When the Youden index was the largest, the predictive cut-off values were 37.0 and 47.9 ms, the sensitivity was 76.3 and 78.9%, and the specificity was 66.7 and 83.3%, respectively (Tables 1, 2). Considering the influence of HR on the QTd index and the better index of the predictive ability between QTd and QTcd, the baseline QTcd served as a useful index of the effectiveness of metoprolol on POTS in children.

### Validation of Predictive Value of Baseline QTcd

In validation group, 24 pediatric POTS (9 boys and 15 girls, mean age of [11.9 ± 2.1] years) who were treated with metoprolol were included. Based on the predictive cut-off value of baseline QTcd by the discovery study, children with POTS in the validation group were divided into QTcd ≥ 47.9 ms group and QTcd <47.9 ms group. Additionally, through follow-up results of the validation group at 3 months after metoprolol treatment, it was found that the sensitivity, specificity, and accuracy of baseline QTcd ≥ 47.9 ms in predicting the effectiveness of metoprolol on pediatric POTS were 73.7, 80.0, and 75.0%, respectively (Table 3).

**Table 3:** Baseline QTcd predicts the efficacy of metoprolol in children with POTS in the validation group (*n* = 24).

**Differential ways to predict the efficacy of metoprolol in children with POTS**		**Clinical standard-based follow-up efficacy outcome**	
		**Responders**	**Non-responders**
Baseline QTcd-based predictive efficacy outcome (QTcd ≥ 47.9 ms)	Responders	14 (73.7%)	1 (20.0%)
	Non-responders	5 (26.3%)	4 (80.0%)

*QTcd, corrected QT interval dispersion; POTS, postural tachycardia syndrome; ms, millisecond*.

## Discussion

We indicated that the baseline QTd and QTcd of the responders were evidently longer than that of the non-responders. Pre-treatment baseline QTd and QTcd were both negatively associated with the post-treatment SS. The baseline QTcd of 47.9 ms had a sensitivity and specificity of 78.9 and 83.3%, respectively, to predict the effectiveness of metoprolol in POTS children. Furthermore, the validation analysis showed that the sensitivity, specificity, and accuracy to predict the effectiveness of metoprolol in children suffering from POTS with a baseline QTcd ≥ 47.9 ms were 73.7%, 80.0%, and 75.0%, respectively. Therefore, baseline QTcd ≥ 47.9 ms could be used as a simple, easy-to-obtain, and inexpensive indicator for predicting the efficacy of metoprolol on pediatric POTS.

Postural tachycardia syndrome is common in children, with an incidence rate of about 6.8%, which seriously damages the physical and psychological health of children, and increases the burden on the family and society (26, 27). Therefore, it was of great significance to give effective intervention on such a disease (28).

The β-adrenergic receptor blockers are important treatment drugs for children with POTS. They play a critical role in blocking the β-adrenergic receptors of the myocardium, but the overall therapeutic effect is not satisfactory (29). The reason includes the complexity and diversity of the pathogenesis of POTS in children, such as overexcitement of sympathetic nerve function, excessive vasodilation, low central blood volume, or endothelial dysfunction (3–6). Metoprolol, as a β-adrenergic receptor blocker, is only effective for POTS children with overexcitement of sympathetic nerve function or high catecholamine status by acting on β1 receptor (30). The patients of POTS with other mechanisms as mentioned above might be the non-responders to the treatment of metoprolol. To find out the useful predictors of the therapeutical response to β-adrenergic receptor blocker, we previously showed several markers to predict the usefulness of β-adrenergic receptor blocker in pediatric POTS (11–14). While, these markers had some limitations, such as the instability, the invasiveness to obtain the samples, the time-consuming or non-economic characteristics, which limits their extensively clinical application. Therefore, it is urgent to explore the non-invasive, easy-to-operate, and inexpensive indexes for the prediction of metoprolol effectiveness for POTS children.

A QT interval is regulated by the autonomic nervous system and the speed of HR. The autonomic nerve exerts an indirect effect on QT interval. When the sympathetic nerve is excited, HR increases and QT interval shortens. While, when the vagus nerve is activated, HR slows down and the QT interval prolongs (31). Cappato et al. reported that vagal tone increased intrinsic dependence of QT at increasing cycle length, whereas sympathetic tone did not seem to interfere significantly (32). Venugopala et al. reported that QTc in healthy children changed little within 24 h, and it was kept constant over a whole day. QTc prolongation indicated impaired cardiac autonomic nerve function and delayed ventricular repolarization (33). QTd in children is increased by the activation of sympathetic nerve or the weakness of vagus nerve. The QTd in children with vasovagal syncope and POTS is significantly higher than that of the healthy children, which indicates that these patients had autonomic nerve dysfunction (31, 34, 35).

Autonomic nerve dysfunction plays an important role in the pathogenesis of pediatric POTS (36–38). It can influence the regulation of autonomic nerves in pediatric POTS. The change of ECG waveform reflects the interaction between the sympathetic nerve and the vagus nerve. QTd reflects the excitability of sympathetic nerves and the release of catecholamines. QTd in children is increased with the activation of sympathetic nerve and the release of excessive catecholamines. The excitement of sympathetic nerve can promote the norepinephrine release that acts on the adrenergic β receptor of the myocardial cell membrane to activate adenylate cyclase (AC)-cyclic adenosine monophosphate (cAMP)-protein kinase A (PKA) (AC-cAMP-PKA) signaling pathway. This phosphorylates the action potential-related ion channels, such as L-type Ca^2+^ channel and K^+^ channels, and then results in the increase of Ca^2+^ influx and acceleration of K^+^ outflow, so that the depolarization and repolarization processes are accelerated and the action potential duration phase is shortened. QTd is referred to the time difference between the earliest repolarization and the latest repolarization of the heart. The distribution of sympathetic nerve in the heart from the bottom to the apex is uneven. As such, when the sympathetic nerve is excited, the value of QT dispersion increases. Therefore, the excessive activation of sympathetic nerve accelerates the Ca^2+^ influx and K^+^ outflow, shortens the time of QT interval, and increases the QTd (36, 37, 39). Therefore, QTd reflects the excitability of sympathetic nerves and the release of catecholamines.

Metoprolol exerts its therapeutic effect on the patients with POTS with the excessive sympathetic nerve function as the main pathogenesis, other than those with different pathogenesis. While QTd obtained by 12-lead ECG is reflective of excessive sympathetic nerve function. Therefore, in the present study, we aimed to examine if the baseline QTd and QTcd could predict the therapeutic effectiveness of metoprolol in children with POTS whose main pathogenesis was the excessive sympathetic nerve function.

QT interval is considered as the sum time of depolarization and repolarization of central ventricular muscle in each cardiac cycle (38). QTd is the difference between QT intervals of each lead on ECG, which refers to the time difference between the earliest repolarization and the latest repolarization of the heart. QTcd is the corrected QTd, a useful index to reflect the cardiac autonomic nerve function, reflecting the asynchrony and electrical instability of ventricular repolarization (40–43). Previous studies on the relationship between QTd and HRV showed that the increased sympathetic or weakened vagal tone could lead to an increased QTd in healthy people (44). In addition, QTd could be increased by enhancing the sympathetic activity and/or reducing vagal activity in the healthy individuals in the process of BHUTT (16). The QTd of children with OI was significantly longer than that of healthy controls (45–47). QTd and QTcd can be obtained by 12-lead ECG, and have the advantages of non-invasiveness, easy-to-operate, and inexpensiveness (48–51).

This study showed that the baseline QTd and QTcd in the responders to metoprolol were significantly longer than the non-responders, indicating that the sympathetic nerve activity and catecholamines release in the responders might be significantly higher than the non-responders. We showed that baseline QTd and QTcd before treatment were negatively correlated with post-treatment SS, indicating that when baseline QTd or QTcd before treatment increased, symptoms would be significantly relieved after metoprolol treatment. On the contrary, if the baseline QTd or QTcd did not increase in POTS of children before treatment, suggesting that the POTS cases did not have marked increase in the sympathetic nerve activity and catecholamines release, the therapeutic effects of metoprolol would not be satisfied. Further analysis showed that the baseline QTcd before treatment successfully predicted the effectiveness of metoprolol in children suffering from POTS. The predictive cut-off value of 47.9 ms yielded the sensitivity and specificity of 78.9 and 83.3%, respectively. Validation studies confirmed that the sensitivity, specificity, and accuracy of a baseline QTcd ≥ 47.9 ms to predict the effectiveness of metoprolol on POTS in children were 73.7, 80.0, and 75.0%, respectively. In conclusion, the baseline QTcd ≥ 47.9 ms before treatment can be regarded as a preliminary clinical indicator of predicting the efficacy of metoprolol in pediatric POTS.

However, this study has certain limitations. It is single-center-based research, and the sample size is not large enough. The specificity and sensitivity of the predictive value of QTd and QTcd are not high enough. Therefore, it would be meaningful to conduct multi-centered and large sample-sized long-term follow-up studies and promote the clinical application of the study results in the future.

## Data Availability Statement

The raw data supporting the conclusions of this article will be made available by the authors, without undue reservation.

## Ethics Statement

The studies involving human participants were reviewed and approved by the Ethics Committee of the Peking University First Hospital. Written informed consent to participate in this study was provided by the participants' legal guardian/next of kin.

## Author Contributions

JD, HJ, CT, YuaW, and YS contributed to conception and design of the study. YuaW, YS, QZ, CZ, and PL contributed to the conduction of the research. YuaW, YS, QZ, CZ, YulW, and PL contributed to the data acquisition and revision of the article for important intellectual content. YuaW contributed to analysis and interpretation of the data. YuaW, YS, HJ, and JD contributed to drafting the article. YuaW, YS, QZ, CZ, PL, YulW, CT, HJ, and JD contributed to final approval of the version to be submitted. All authors contributed to the article and approved the submitted version.

## Funding

This study was supported by the Science and Technology Program of Beijing (Z171100001017253).

## Conflict of Interest

The authors declare that the research was conducted in the absence of any commercial or financial relationships that could be construed as a potential conflict of interest.

## Publisher's Note

All claims expressed in this article are solely those of the authors and do not necessarily represent those of their affiliated organizations, or those of the publisher, the editors and the reviewers. Any product that may be evaluated in this article, or claim that may be made by its manufacturer, is not guaranteed or endorsed by the publisher.

## Abbreviations

QTd: QT interval dispersion
QTc: corrected QT interval
QTcd: corrected QT interval dispersion
POTS: postural tachycardia syndrome
ECG: electrocardiogram
SS: symptom score
OI: orthostatic intolerance
HR: heart rate
CNP: C-type natriuretic peptide
HRV: heart rate variability
BP: blood pressure
HUTT: head-up tilt test
AC: adenylate cyclase
cAMP: cyclic adenosine monophosphate
PKA: protein kinase A.
